# Targeted Delivery of Paclitaxel in Liver Cancer Using Hyaluronic Acid Functionalized Mesoporous Hollow Alumina Nanoparticles

**DOI:** 10.1155/2019/2928507

**Published:** 2019-04-15

**Authors:** Yu Gao, Lili Hu, Ying Liu, Xiaoyan Xu, Chao Wu

**Affiliations:** ^1^Department of Medical Oncology, The First Affiliated Hospital of Jinzhou Medical University, No.2, The Fifth Section of Renmin Street, Guta District, Jinzhou, Liaoning Province 121001, China; ^2^Department of Pharmaceutics, Jinzhou Medical University, 40 Songpo Road, Linghe District, Jinzhou, Liaoning Province 121001, China

## Abstract

Hyaluronic acid functionalized mesoporous hollow alumina nanoparticles (HMHA) were used as a tumor-targeted delivery carrier for liver cancer therapy. Paclitaxel (PAC) incorporated in the carrier by the adsorption method was analyzed by X-ray diffraction and differential scanning calorimetry. PAC was found to be in an amorphous state. The hyaluronic acid coated on the surface of mesoporous hollow alumina nanoparticles (MHA) regulated the drug release rate and the loaded samples obtained a sustained drug release. In vitro experiments demonstrated that paclitaxel-hyaluronic acid functionalized mesoporous hollow alumina nanoparticles (PAC-HMHA) had a high cellular uptake, which increased the drug level in tumor tissues and was beneficial to promote apoptosis. An in vivo tumor inhibition rate study demonstrated that PAC-HMHA (64.633 ± 4.389%) had a better antitumor effect than that of paclitaxel-mesoporous alumina nanoparticles (PAC-MHA, 56.019 ± 6.207%) and pure PAC (25.593 ± 4.115%). Therefore it can be concluded that PAC-HMHA are a prospective tumor-targeted delivery medium and can be useful for future cancer therapy.

## 1. Introduction

Liver cancer is a lethal disease with high incidence and requires particular attention. Traditional chemotherapy, with high cytotoxicity in various tissues, is perplexing. Currently, novel nanodrug delivery systems have been studied in order to obtain a better therapy for liver cancer and these systems include polymeric nanoparticles [1, 2], liposomes [3, 4], and inorganic material nanoparticles [5–8]. These forms can improve the drug circulation time in the blood and passively target tumor tissues, due to the enhanced permeability and retention effect (EPR). Although passive targeting reduces the side effects of chemotherapeutic drugs, the drug concentration in the tumor cells still is insufficient. For effective targeting, various liver-targeting ligands have been grafted to the surface of the carriers to overcome the problem of low target efficiency. Commonly used ligands include folic acid, galactose, protein, hyaluronic acid (HA), and glycyrrhetinic acid [9–13]. Ligand-modified nanodrug delivery systems can enter tumor tissues by receptor-mediated endocytosis and obtain the optimum antitumor effect.

Recently, HA with high targeting efficiency, good biocompatibility, good biodegradability, and nontoxicity can bind specifically with cluster determinant 44 (CD44) receptors on the hepatoma cell membrane, which is widely applied in the functionalization of carrier materials. Moreover, with the development of nanomaterials, metallic oxides excite researchers for use as antitumor drug carriers, such as zinc oxide nanoparticles [14], copper oxide nanowires [15], alumina nanoparticles [16], and ferric oxide nanoparticles [17]. Their nanostructure characteristics show unique potential as a carrier material. Among them, alumina, with extensive biological application potential, attracts our attention [18–22]. Zhao J. et al. made Vx3-functionalized alumina nanoparticle-loaded ubiquitinated proteins for improving cancer immunotherapy [23]. Xifreperez E. et al. applied bovine serum albumin- (BSA-) functionalized porous alumina particles as a carrier and investigated the early diagnosis and targeted treatment for HepG2 tumor cells [24]. Wang Y. et al. prepared anodic alumina nanotubes loaded with the proapoptotic protein apo2L/TRAIL for use as a potential drug carrier for human breast cancer therapy [25]. Owing to good performance of alumina materials, it is a suitable antitumor vector.

In this study, mesoporous hollow alumina nanoparticles (MHA) were prepared by the template method and mesoporous carbon nanoparticles were used as the template material. After the carrier was aminated, HA was grafted with the amino group of MHA for better targeting efficiency. The mesoporous hollow structure of MHA was suitable for drug storage. PAC was integrated with HMHA as a first-line chemotherapeutic drug. The inhibitory and antitumor effects of the prepared PAC-HMHA were analyzed in vitro using liver cancer cells and in vivo using tumor-bearing nude mice.

## 2. Materials and Methods

### 2.1. Materials

Paclitaxel (PAC) was supplied by Tianfeng Biotechnology Company (Xian, China). Tetrapropyl orthosilicate (TPOS), (3-aminopropyl)triethoxysilane (APTES), N-hydroxysuccinimide(NHS), and 1-ethyl-3-(3-dimethylaminopropyl)carbodiimide (EDC) were purchased from Aladdin reagent company. Ethanol, NH_3_H_2_O, resorcinol, formaldehyde, hydrofluoric acid, Al(NO_3_)_3_, hyaluronic acid (HA), methyl tert-butyl ether, chloroform, acetonitrile, fetal bovine serum (FBS), RPMI 1640 medium, 3-(4,5-dimethylthiazol-2-yl)-2,5-diphenyltetrazolium (MTT), dimethyl sulfoxide (DMSO), propidium iodide (PI), Annexin V-FITC, and trypsin were bought from Beijing Dingguo Changsheng Biotech Co., Ltd. (Beijing, China).

### 2.2. Preparation of HMHA

#### 2.2.1. Synthesis of Particles

Mesoporous carbon hollow nanospheres were synthesized according to the previous reports [26]. Briefly, TPOS (3.46 mL) was dripped into a mixed solution composed of ethanol (70 mL), NH_3_H_2_O (3 mL), and H_2_O (10 mL) under stirring for 15 min, and then resorcinol (0.4 g) and formaldehyde (0.56 mL) were added to the above solution. The reaction lasted 24 h under stirring. The precipitates obtained by centrifugation were dried at 50°C and then calcined at 700°C under a N_2_ atmosphere for 7 h. To remove the silica, 5 wt% of hydrofluoric acid was used. The obtained product was mesoporous carbon hollow nanospheres (MCHN), of which, 100 mg was dispersed in 10 mL of Al(NO_3_)_3_ solution (1.5 M) under stirring. After 24 h, the MCHN-Al(NO_3_)_3_ samples obtained by centrifugation were dried at 50°C and then calcined at 500°C under an air atmosphere for 3 h. The product was mesoporous hollow alumina nanoparticles (MHA). Amino-functionalized MHA (MHA-NH_2_) was obtained by reacting with APTES [27]. A certain amount of NHS and EDC was added to 70 mL of HA solution (2 mg/mL) under stirring for activating carboxyl of HA. Two hundred milligrams of amino-functionalized MHA was added to the above HA solution. The system was stirred for 12 h. The product (HMHA) obtained by centrifugation was dehydrated with ethanol and then dried under a vacuum.

### 2.3. Drug Loading

The adsorption equilibrium method was used to load PAC into HMHA [28]. Briefly, 100 mg of HMHA was dispersed in 100 mg/mL of PAC chloroform solution under stirring for 5 h. The obtained samples by centrifugation were dried under a vacuum. The drug loading of MHA is the same as the above process. Paclitaxel-mesoporous alumina nanoparticles (PAC-MHA) were used for comparison. High-performance liquid chromatography (HPLC, LC-2030, Shimadzu, Japan) was applied to determine the drug loading. The mobile phase was a solution composed of acetonitrile and water with a ratio of 50:50 (v/v). The drug loading (DL) of HMHA was obtained by the following equation.

(1) $$\begin{array}{c} DL\;\left(\;\%\;\right)=\frac{\text{PAC amount in PAC-HMHA}}{\text{PAC-HMHA amount}} \end{array}$$

### 2.4. Characterization

Transmission electron microscopy (TEM, Tecnai G2F30, FEI, USA) was employed to observe the morphological structure characteristics of carriers. The HA content in HMHA was estimated by thermogravimetric analysis (TGA). The equipment used was a TGA-50 instrument (Shimadzu, Japan). The current state of the drug was observed under nitrogen using a differential scanning calorimeter (DSC-60, Shimadzu, Inc. Japan). The X-ray diffraction (XRD) patterns of the samples were obtained using an X-ray diffractometer (Rigaku Denki, Japan) with a range (2*θ*) from 3°C to 60°C. Fourier transform infrared spectroscopy (FTIR) was performed by FTIR spectrometry (Bruker IFS 55, Switzerland).

### 2.5. Drug Release

The release test was performed by the paddle method (RC806D, Tianda Tianfa, China). The samples (equivalent to 3 mg PAC) were dispersed in 400 mL of PBS release medium with pH 7.4. Drug release medium satisfied sink conditions. The speed and temperature were 100 rpm and 37°C. At 5 min, 10 min, 15 min, 20 min, 30 min, 45 min, 1 h, 2 h, 4 h, 6 h, 12 h, and 24 h, 2 mL of release medium was taken and centrifuged at 10000 rpm for 10 min. Then, the PAC concentration was determined by HPLC.

### 2.6. Cell Viability Assays

The cytotoxicity assays of HMHA and the loaded samples were performed with liver cancer SMMC-7721 cell line. The counted cells were cultured in 96-well plates under a CO_2_ atmosphere. The culture medium used was RPMI 1640 medium containing 10% FBS. After 24 h, the cells adhered to the wall and the medium was replaced with sample suspensions with the different concentrations, which were composed of samples, RPMI 1640 medium, FBS, and 2% hypromellose. After 48 h of cultivation, the sample suspension was removed and 20 *μ*L of MTT (5 mg/mL) solution was added. The cells were further incubated for 4 h. DMSO (200 *μ*L) replaced the above medium, and then the absorbance was determined by microplate reader at 492 nm after 10 min of shaking.

### 2.7. Apoptosis Assay

Flow cytometry (Becton Dickinson, Zürich, Switzerland) was used to record the cell apoptosis rate. The counted cells were cultured in 6-well plates under a CO_2_ atmosphere. The cells adhered to the wall and then PAC suspensions, PAC-MHA suspensions, and PAC-HMHA suspensions (correspond to 10 ng/mL of PAC concentration) replaced the culture medium. After incubation for 48 h, the cells were digested by trypsin. The above medium was replaced by 500 *μ*L of binding buffer, and then the cells were stained using Annexin V-FITC (5 *μ*L) and PI (5 *μ*L). The apoptotic cells were determined by flow cytometry.

### 2.8. Cellular Uptake

The fluorescent labeling process of the HMHA was as follows. A certain amount of HMHA was dispersed in an FITC ethanol solution (2 mg/mL) and the system was stirred for 5 h. The isothiocyanato group of FITC could bind with the amino group on the inner surface of the HMHA channel. The labeling process of MHA with the amino group was the same as that described above. FITC-labeled MHA was used as a contrast. FITC-labeled HMHA was obtained and used to observe the cellular uptake of HMHA. The counted cells were cultured in a confocal culture dish under a CO_2_ atmosphere for 24 h. FITC-labeled HMHA suspensions (the equivalent of 50 *μ*g/mL PAC) were used to treat the cells for 0.5 h, 1 h, and 1.5 h, and then 4% paraformaldehyde solution fixed the cells. After staining by Hoechst 33342 and rhodamine B, the cells were observed by confocal laser scanning microscopy. The process of FITC-labeled MHA was the same as that described above.

In order to further quantify the PAC uptake efficiency, the counted cells were cultured in culture bottles for 24 h. The PAC suspension, PAC-HMHA suspension, and PAC-MHA suspension (the equivalent of 50 *μ*g/mL PAC for PAC-HMHA and PAC-MHA) were used to treat the cells for 0.5 h, 1 h, and 1.5 h. After the cells were collected, they were lysed using ultrasonicator to extract the drug. Methyl tert-butyl ether, as the extraction solvent, was evaporated, and then the obtained dried samples were redissolved in the mobile phase. HPLC was used to analyze the drug concentration.

### 2.9. Antitumor Efficacy

The antitumor efficacy of PAC-HMHA was checked using a nude mouse model with liver cancer SMMC-7721 cells (Beijing Weitong Lihua Experimental Animal Technology Co. Ltd.). Jin Zhou Medical University Laboratory Animal Ethics Committee (No. 11400700217882) authorized the experiment. In this experiment, 5 × 10^4^ SMMC-7721 cells were injected subcutaneously into the right front legs of nude mice. After the tumor volume reached 50–70 mm^3^, 20 mice were randomly placed into four groups. Physiological saline, PAC, PAC-MHA, and PAC-HMHA suspension were injected intraperitoneally; the dose was 20 mg/kg and the time interval was 3 days. A caliper was used to measure the tumor volume before each administration. The equation was V = 0.5 × length × (width)^2^. Tumor tissues were excised after 5 dose cycles and fixed with paraformaldehyde. Paraffin-embedded tumor specimens were sliced and stained with hematoxylin and eosin (H&E). The obtained tumor sections were surveyed by fluorescence microscopy (Leica DMI 4000B, Germany).

### 2.10. Statistical Analysis

The mean values plus standard deviations were used to show the parameter values. Statistical analysis testing (ANOVA and Bonferroni tests) using SPSS 17.0 software was carried out for all resulting values, and the results were found to be statistically significant.

## 3. Results and Discussion

### 3.1. Carrier Morphological Structure and Drug Loading

**Scheme 1:**
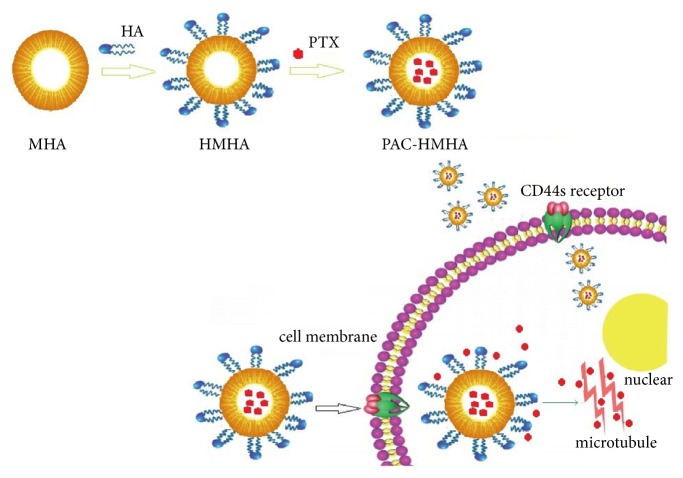
Schematic illustration of the preparation process and action mechanism of PAC-HMHA. MHA, mesoporous hollow alumina nanoparticles; HMHA, hyaluronic acid functionalized mesoporous hollow alumina nanoparticles; PAC-HMHA, paclitaxel-hyaluronic acid functionalized mesoporous hollow alumina nanoparticles.

**Figure 1:**
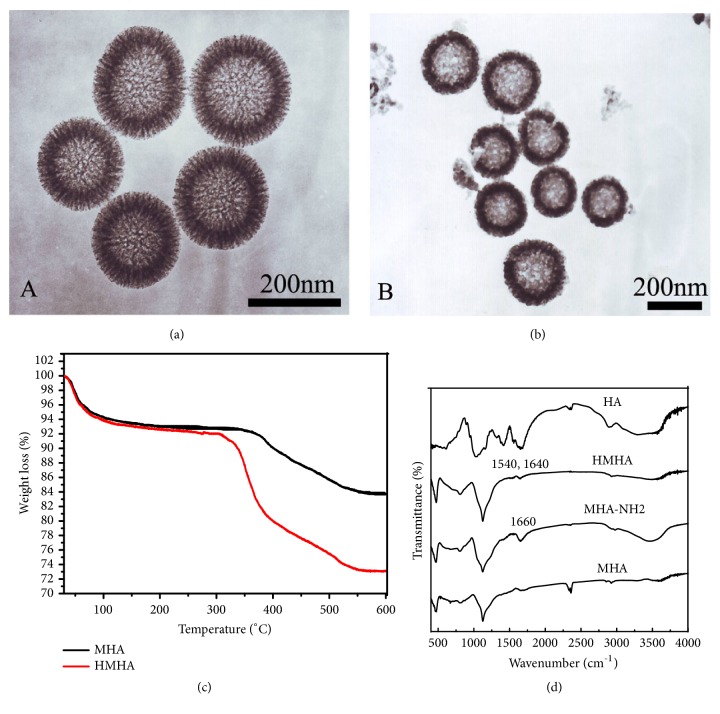
TEM of (a) MCHN and (b) MHA; (c) TGA characterization; (d) FTIR characterization. MCHN, mesoporous carbon hollow nanospheres; MHA, mesoporous hollow alumina nanoparticles; MHA-NH_2_, amino-functionalized MHA; HMHA, hyaluronic acid functionalized mesoporous hollow alumina nanoparticles; HA, hyaluronic acid.

The preparation process and action mechanism of PAC-HMHA are clearly shown in Scheme 1. The hard-template method, with advantages including a simple and scalable process, monodispersed morphology, and adjustable particle size, was used for synthesizing MHA. A mesoporous carbon hollow nanosphere (MCHN) was prepared first as the hard template. Aluminum nitrate was adsorbed into the mesoporous structure of MCHN, and then MHA was obtained by high-temperature calcination followed by modification of the surface of the nanoparticles with the amino group. The carboxyl group of HA covalently cooperated with the amino group on the surface of the MHA. The obtained HMHA was loaded with PAC by the adsorption method. The PAC-HMHA can be efficiently incepted into the cell because HA can be specifically recognized and combined with the CD44 receptor on the surface of the cell membrane [29]. In our study, MCHN and MHA were successfully prepared. As shown in Figure 1(a), the TEM image of MCHN had a mesoporous wall and hollow structure. The particle size was approximately 200 nm. The TEM image of MHA in Figure 1(b) displayed the mesoporous hollow structure, which was successfully replicated from the MCHN. In order to verify the cladding of HA, a TGA diagram (Figure 1(c)) showed that HMHA presented a weight loss of approximately 6% compared with amino-functionalized MHA (MHA-NH_2_), which explained the covered HA amount. The FTIR spectra results in Figure 1(d) indicate that MHA-NH_2_ had a NH_2_ bending vibration peak at 1660 cm^−1^. In HMHA, 1540 cm^−1^ (NH in-plane bending vibration) and 1640 cm^−1^ (amide bond) indicated that the HA successfully bonded with the amino groups. The drug loading of PAC-HMHA was 29.45 ± 2.16%, whereas that of PAC-MHA was 35.21 ± 1.91%. The pore volume of the carrier decreased due to the coating of HA, which induced a reduction in drug loading. The above results showed that HMHA was suitable for drug storage.

### 3.2. XRD and DSC Analysis

**Figure 2:**
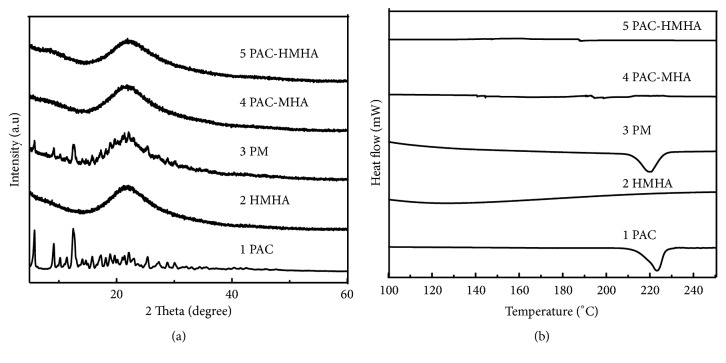
The XRD (a) and DSC (b) characterization of PAC, HMHA, PM, PAC-MHA, and PAC-HMHA. PAC, paclitaxel; HMHA, hyaluronic acid functionalized mesoporous hollow alumina nanoparticles; PM, the physical mixture of the drug and HMHA; PAC-MHA, paclitaxel-mesoporous hollow alumina nanoparticles; PAC-HMHA, paclitaxel-hyaluronic acid functionalized mesoporous hollow alumina nanoparticles.

Figure 2(a) shows the XRD of PAC, HMHA, the physical mixture (PM, the ratio of the drug and HMHA was the same as that of the loaded samples), PAC-MHA, and PAC-HMHA. The XRD results for HMHA was amorphous. PAC and PM displayed the crystalline state of drugs at 5.8° and 12.5°. By comparison, PAC-MHA and PAC-HMHA did not present the crystalline characteristics of PAC. The above results indicated that PAC in the mesoporous channel of MHA or HMHA was amorphous, which was confirmed by DSC analysis. DSC curves of PAC, HMHA, PM, PAC-MHA, and PAC-HMHA are shown in Figure 2(b). The DSC for HMHA did not show a phase transformation, which indicates that HMHA were amorphous. PAC and PM displayed a phase transformation of the drug at 223°C. By comparison, PAC-MHA and PAC-HMHA did not present the crystal-phase transformation of PAC at 223°C. Finally, we identified that PAC was in an amorphous state, which was the direct reason for the improvement in the drug's solubility.

### 3.3. In Vitro Release

**Figure 3:**
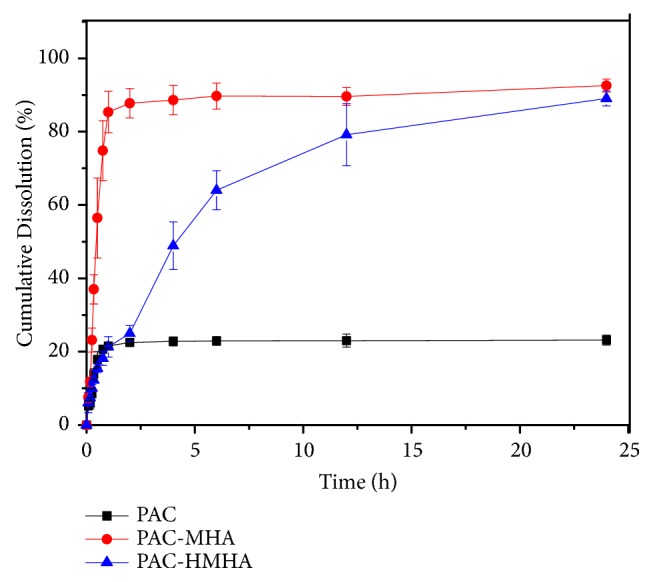
In vitro release curves of PAC, PAC-MHA and PAC-HMHA. Data represented as mean ± SD (n=3). PAC, paclitaxel; PAC-MHA, paclitaxel-mesoporous hollow alumina nanoparticles; PAC-HMHA, paclitaxel-hyaluronic acid functionalized mesoporous hollow alumina nanoparticles.

The greatly enhanced dissolution by MHA and regulated release rate by HMHA indicated high drug release in vitro. Therefore, comparative evaluation of the in vitro release of PAC-MHA and PAC-HMHA was carried out in the release test. In Figure 3, compared with 21.59 ± 1.15% release of PAC at 1 h, PAC-MHA significantly improved drug dissolution and reached 85.35 ± 3.25% at 1 h. This indicated that the mesoporous hollow structure of MHA was highly dispersed in PAC and the amorphous state of PAC was the cause of improvement in the drug dissolution. PAC-HMHA exhibited the effect of sustained release, and the dissolution amount reached 89.03 ± 2.79% at 24 h. The cladding of HA increased the drug diffusion resistance, which led to the production of the sustained release effect. This could increase the drug absorption and avoid the recrystallization caused by rapid dissolution of PAC.

### 3.4. In Vitro Cytotoxicity and Apoptosis Analysis

**Figure 4:**
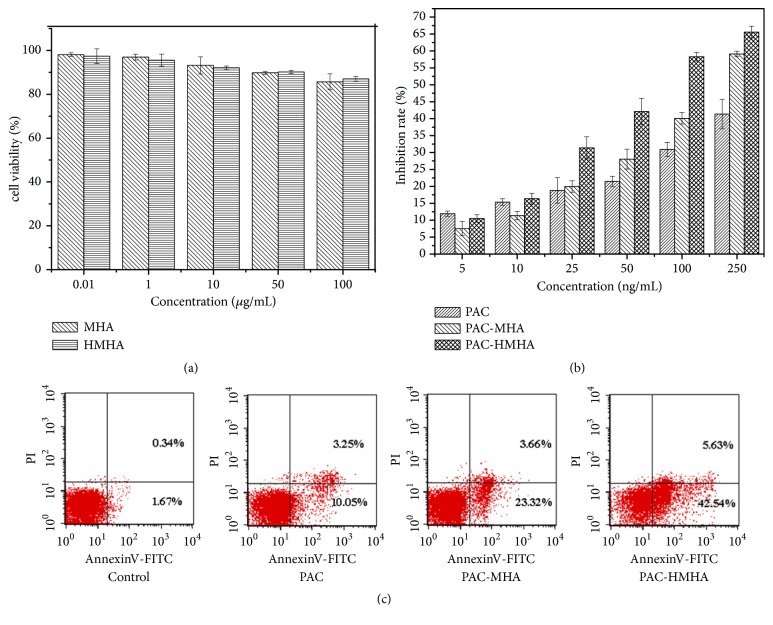
Cell viability (a) of MHA, HMHA; cell inhibition (b) and apoptosis analysis (c) of PAC, PAC-MHA, and PAC-HMHA. Data represented as mean ± SD (n=6). MHA, mesoporous hollow alumina nanoparticles; HMHA, hyaluronic acid functionalized mesoporous hollow alumina nanoparticles; PAC, paclitaxel; PAC-MHA, paclitaxel-mesoporous hollow alumina nanoparticles; PAC-HMHA, paclitaxel-hyaluronic acid functionalized mesoporous hollow alumina nanoparticles.

As shown in Figure 4(a), both MHA and HMHA at a maximum concentration of 100 *μ*g/mL showed more than 90% cell viability, which demonstrated that MHA and HMHA for SMMC-7721 cell had good biosafety. In Figure 4(b), compared with PAC-MHA and PAC, PAC-HMHA had higher inhibition rate at the 5–250 ng/mL concentration range for SMMC-7721 cells. At 250 ng/mL of PAC concentration, cell viability of PAC group was 58.6 ± 2.39%. In comparison, PAC-MHA and PAC-HMHA showed 40.9 ± 1.83% and 34.4 ± 2.04% cell viability, respectively. The IC50 of PAC, PAC-MHA, and PAC-HMHA were 1022.57 ± 15.79% ng/mL, 174.41 ± 7.48% ng/mL, and 77.46 ± 5.26% ng/mL, respectively. All these results illustrated that PAC-HMHA effectively inhibits the proliferation of tumor cells. The nanoscale effect of HMHA and targeting effect of HA could increase the drug accumulation in tumor cells, which leads to high drug concentrations in tumor cells. This was the primary reason for strongly inhibiting cell proliferation [30]. The apoptosis analysis confirmed the above conclusion. PAC-HMHA in Figure 4(c) induced cell early apoptosis in 42.54 ± 3.19% of SMMC-7721 cells, in comparison with 10.05 ± 1.53% by PAC-MHA and 23.32 ± 1.67% by PAC at 10 ng/mL of PAC concentration, indicating that PAC-HMHA effectively promoted apoptosis of SMMC-7721 cells. Therefore, HMHA have a targeted effect on SMMC-7721 cells.

### 3.5. Cellular Uptake Analysis

**Figure 5:**
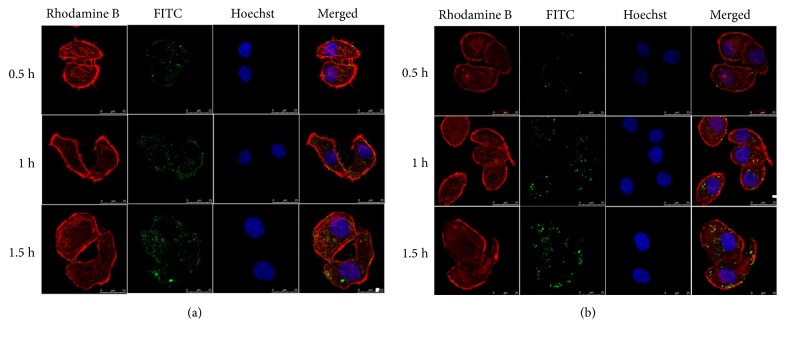
LSCM images of SMMC-7721 cell line treated with FITC-labeled HMHA (a) and FITC-labeled MHA (b) at 0.5 h, 1 h, and 1.5 h. MHA, mesoporous hollow alumina nanoparticles; HMHA, hyaluronic acid functionalized mesoporous hollow alumina nanoparticles.

**Figure 6:**
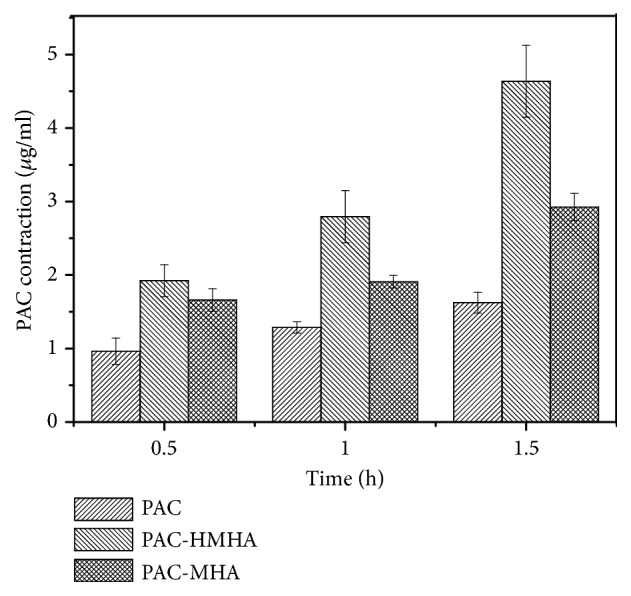
The cellular uptake of PAC, PAC-MHA, and PAC-HMHA from HPLC. PAC, paclitaxel; PAC-MHA, paclitaxel-mesoporous hollow alumina nanoparticles; PAC-HMHA, paclitaxel-hyaluronic acid functionalized mesoporous hollow alumina nanoparticles.

In order to verify the increased drug accumulation in tumor cells, a cellular uptake test was performed and the results are shown in Figure 5(a). FITC-labeled HMHA at 0.5 h were observed in cells by green fluorescence, which demonstrated that the carriers were taken up by tumor cells. With increasing incubation time, the intake amount significantly increased according to the changes in the green fluorescence and tumor cells for the uptake of HMHA, indicating time dependence. Moreover, as a comparison, FITC-labeled MHA had a significantly lower intake at the same time, as shown in Figure 5(b). This indicates that the coated HA had an obvious targeting effect. The HPLC results in Figure 6 were consistent with the above results. At 0.5 h, the concentration of PAC in the PAC-MHA group was 0.963 ± 0.179 *μ*g/mL, compared with 1.662 ± 0.154 *μ*g/mL of the PAC group. The PAC-HMHA group had a higher concentration (1.924 ± 0.216 *μ*g/mL). There is statistical significance (P<0.05) between PAC-HMHA group and PAC-MHA group according to ANOVA and Bonferroni tests. That indicates that the coating of HA enables outstanding targeting of MHA. As time went on, the uptake of PAC also increased significantly in each group, which indicates that the cellular uptake of PAC is time-dependent. The above conclusion will lead to high drug concentration in cells, which will speed up cell apoptosis.

### 3.6. Antitumor Effect in Solid-Tumor-Bearing Nude Mice

**Figure 7:**
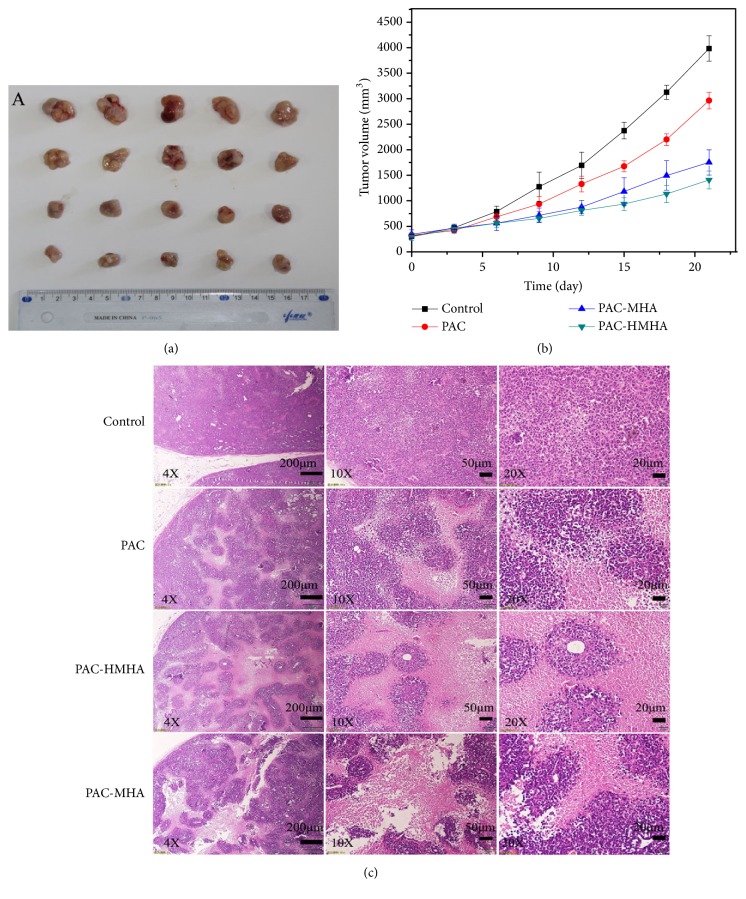
(a) The tumor tissues images from the tumor-bearing nude mice of physiological saline, PAC, PAC-MHA, and PAC-HMHA groups. (b) The tumor volume curves. (c) Histological examination of excised tumor tissues under different magnification. PAC, paclitaxel; PAC-MHA, paclitaxel-mesoporous hollow alumina nanoparticles; PAC-HMHA, paclitaxel-hyaluronic acid functionalized mesoporous hollow alumina nanoparticles.

To test whether the antitumor effect in vivo is as good as that in cell experiments, a comparative study of physiological saline, PAC, PAC-MHA, and PAC-HMHA suspension was performed, and the results are shown in Figures 7(a) and 7(b). The PAC-HMHA group (64.633 ± 4.389%) had a better effect at inhibiting the tumor volume than the PAC group (25.593 ± 4.115%) and the PAC-MHA group (56.019 ± 6.207%). The results can be attributed to the accumulation of PAC-HMHA in the tumors due to the nanoscale effect and targeting action of HMHA. The histological photomicrographs of the excised tumor tissues in Figure 7(c) show that the PAC-HMHA group had a more significant necrotic region compared to the PAC group and the PAC-MHA group. The shrinkage and fragmentation of the nuclei was also more visible. In summary, the PAC-HMHA group had a better antiproliferation effect.

## 4. Conclusion

PAC-HMHA was obtained and used to treat liver cancer. In vitro cell experiments indicated that PAC-HMHA could be ingested by SMMC-7721 cells and prompt cell apoptosis. Compared with PAC and PAC-MHA, PAC-HMHA showed a good tumor-suppression effect in vivo. Overall, this study indicated that HMHA exhibits tumor control ability and is a potential candidate for the development of carrier materials.

## Data Availability

All data generated included in this published article are available from the corresponding author on reasonable request.
